# Comparative analysis of operative cost in lung resection: preoperative robotic navigational bronchoscopy (Nav) vs. intraoperative wedge biopsy

**DOI:** 10.1007/s11701-026-04170-2

**Published:** 2026-11-10

**Authors:** Sneha S. Alaparthi, Gabrielle Kozlowski, Isheeta Madeka, Brian Mitzman, John Jacob, Tyler Grenda, Nathaniel Evans, Olugbenga T. Okusanya

**Affiliations:** 1 Department of Surgery, Thomas Jefferson University Hospital, 1015 Walnut Street Curtis Building Suite 620, Philadelphia, PA 19107 USA; 2 Sidney Kimmel Medical College, Thomas Jefferson University, Philadelphia, PA USA; 3 Division of Cardiothoracic Surgery, University of Utah and Huntsman Cancer Institute, Salt Lake City, United States

**Keywords:** Lung resection, Robotic surgery, Cost, Frozen section, Biopsy, Bronchoscopy

## Abstract

Evaluate differences in operative cost in pulmonary resection between patients who had preoperative biopsy via robotic navigational bronchoscopy versus an intraoperative diagnostic wedge. We performed a retrospective cohort study of patients who underwent standard lobectomy at our institution between January 2024 and July 2025. We identified patients who had a preoperative robotic navigational bronchoscopy (Nav) prior to lung resection and those who had no preoperative biopsy (NPB) and had intraoperative diagnostic wedge. We then examined operative time, stapler load quantity, stapler cost, robotic cost, and overall operative cost. 128 patients were identified, 53 in the NPB cohort and 75 in the Nav cohort. The mean age of the cohort was 68. The average surgical cost of the non-preoperative biopsy (NPB) cohort was $10,258.68 and $9,249.36 in the Nav cohort (*P* = 0.007). The average stapler loads used, total stapler cost, total robotic I&A costs, and surgical supply costs were higher in the NPB cohort (*P* < 0.001). The average technical cost of performing a frozen specimen was $65.35. There was no statistically significant difference in operative time between cohorts, however the NPB cohort had a longer operative time on average (187.83 min vs. 174.63 min, *P* = 0.161). We found that on average, surgical cost, operative time, robotic cost, and OR charges were higher in patients who had undergone intraoperative frozen section (NPB) compared to those with preoperative biopsy (Nav). This data suggest that the use of preoperative robotic bronchoscopy is associated with decreased cost of robotic lung resection.

## Introduction

Surgical resection remains a mainstay for early-stage lung cancer. As such, new diagnostic techniques, such as navigational bronchoscopy (Nav), have emerged to improve quality of staging and resection. This technique of bronchoscopy involves a robotic system with an articulating catheter which is guided into distal airways to access a lesion in real time. This technique has become a growing option in the field of lung cancer diagnostics, and has proven to be effective, accurate, and safe when compared to traditional CT guided approach [1, 2].

There remains a subject of patients, however, which upfront resection without biopsy is recommended. These are cases in which index of suspicion for malignancy is high, or for small peripheral lung nodules which may not be accessible by CT or bronchoscopy [3]. In this case, a wedge resection followed by an anatomic lung resection if malignancy is confirmed remains an appropriate approach. This technique remains highly sensitive and specific for the diagnosis of malignancy from lung nodules, and is well correlated with final pathology results [3, 4]. For many, navigational bronchoscopy or intraoperative wedge or even diagnostic lobectomy is the preferred approach for local, patient related and resource-related reasons.

As lung cancer remains a leading cause of cancer death worldwide, it is imperative to not only examine effectiveness of diagnostic techniques, but also financial implications. There remains limited data on cost of both preoperative Nav bronchoscopy and surgical resection with intraoperative biopsy. No research to date has compared intraoperative costs in these populations as well.

In this study we aim to examine patients at a single institution who underwent lung resection and had a preoperative navigational bronchoscopy (nav) or an intraoperative frozen section. We plan to examine costs of surgical resection between these cohorts. Our hypothesis is that for patients undergoing preoperative biopsy with navigational bronchoscopy, operative costs will be lower.

## Materials and methods

This is a retrospective cohort study of patients who underwent uncomplicated lung resection at Thomas Jefferson University Hospital (TJUH) between January 2024 and July 2025. This study was deemed exempt by the Institutional Review Board (IRB) at TJUH (IRB # 2024 − 1235). Data was collected using manual chart review. We identified patients who underwent robotic navigational bronchoscopy (Nav) or no preoperative biopsy (NPB, intraoperative frozen section). Any patients who had other means of biopsy (i.e. traditional bronchoscopy, CT guided biopsy) were excluded. Patients with a non-diagnostic Nav result who did not undergo frozen section were excluded. Benign pathologies were excluded. Patients in the NPB cohort who did not undergo anatomic lung resection after frozen wedge were excluded. Patients with incomplete or missing data were excluded from the final analysis.

Demographic data such as age, race and sex were extracted from the chart. Data on the lung nodule such as cytology, final pathology and number of frozen specimens was examined. We then collected the following variables surrounding cost and operative details: cost of frozen specimen, type of stapler used, cost of stapler loads used, total stapler cost, robotic instruments and accessories (I&A) cost, operative time in minutes, operative cost per minute, surgical supply cost, and total combined surgery cost.

Statistical analysis was performed using SPSS. Mean, median, and averages were calculated. Cohorts were compared using a Student’s T- test. A P value < 0.05 was considered statistically significant. Categorical variables, including clinical stage, procedure type (frozen section biopsy vs. navigational bronchoscopy), sex, and race/ethnicity, were summarized as frequencies and percentages.

## Results

A total of 128 patients were identified. 89 patients were female (69.5%), and 39 male (30.5%). Among the full cohort, 94 patients were white (73.4%), followed by 22 Black (17.2%), 6 Asian (4.7%), and 5 Hispanic (3.9%). The average age of all patients was 67.97 (Table 1).

**Table 1:** Demographics

Characteristic	Overall (*n* = 128)	Frozen (*n* = 53)	Nav (*n* = 75)	*p*-value
**Age**,** years (mean ± SD)**	68.0 ± 8.3	67.2 ± 8.8	68.5 ± 7.9	0.3722
**Gender**,** n (%)**				0.5982
Female	89 (69.5%)	35 (66.0%)	54 (72.0%)	
Male	39 (30.5%)	18 (34.0%)	21 (28.0%)	
**Race/Ethnicity**, n (%)*				0.2007
Asian	4 (3.1%)	1 (1.9%)	3 (4.0%)	
Asian/Chinese	2 (1.6%)	0 (0.0%)	2 (2.7%)	
Black/African American	22 (17.2%)	11 (20.8%)	11 (14.7%)	
Hispanic	5 (3.9%)	4 (7.5%)	1 (1.3%)	
White/Caucasian	94 (73.4%)	36 (67.9%)	58 (77.3%)	

*One patient with missing information, declined to report race/ethnicity

**Fig. 1:**
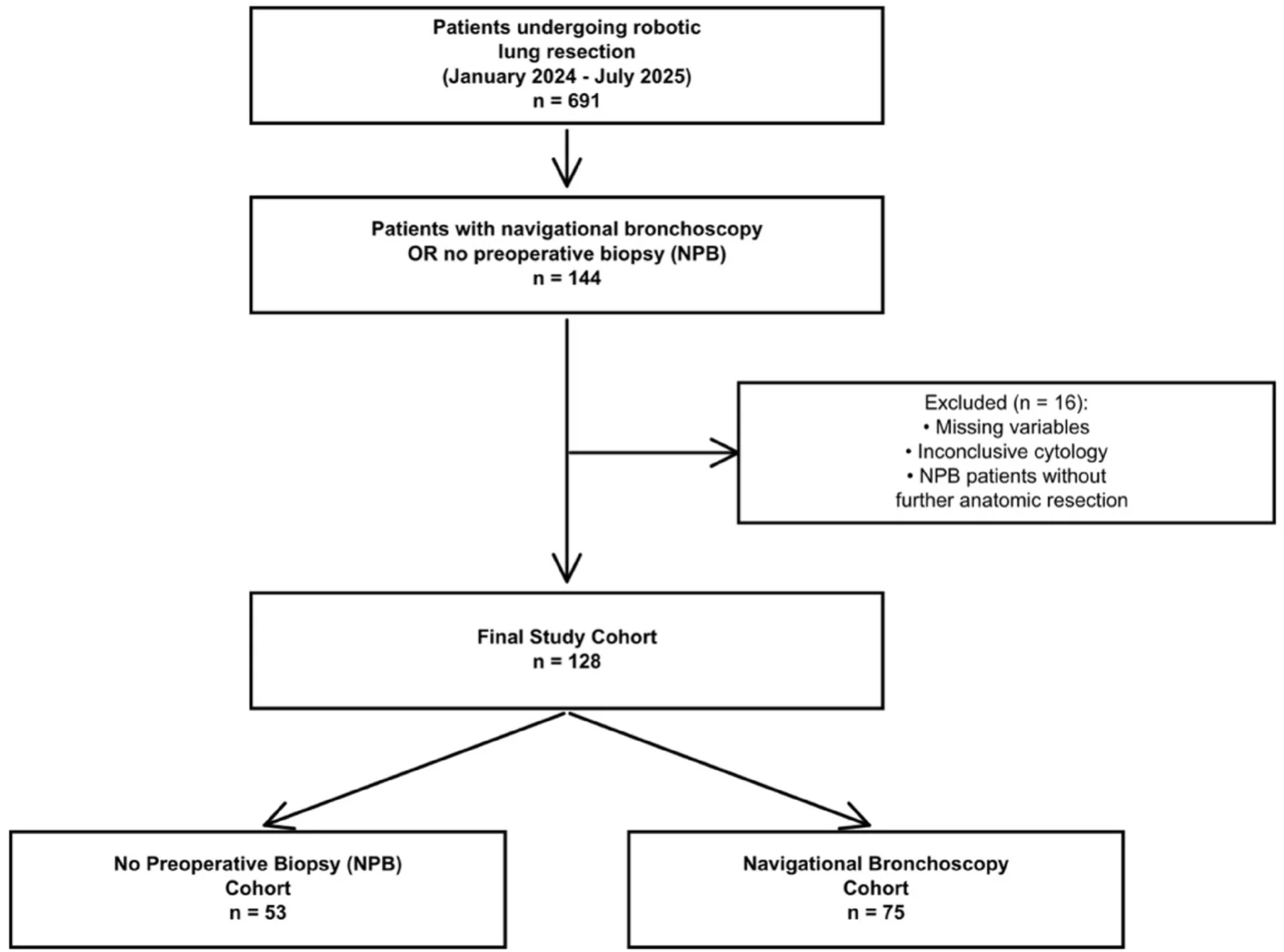
Patient Selection Flow Diagram

**Fig. 2:**
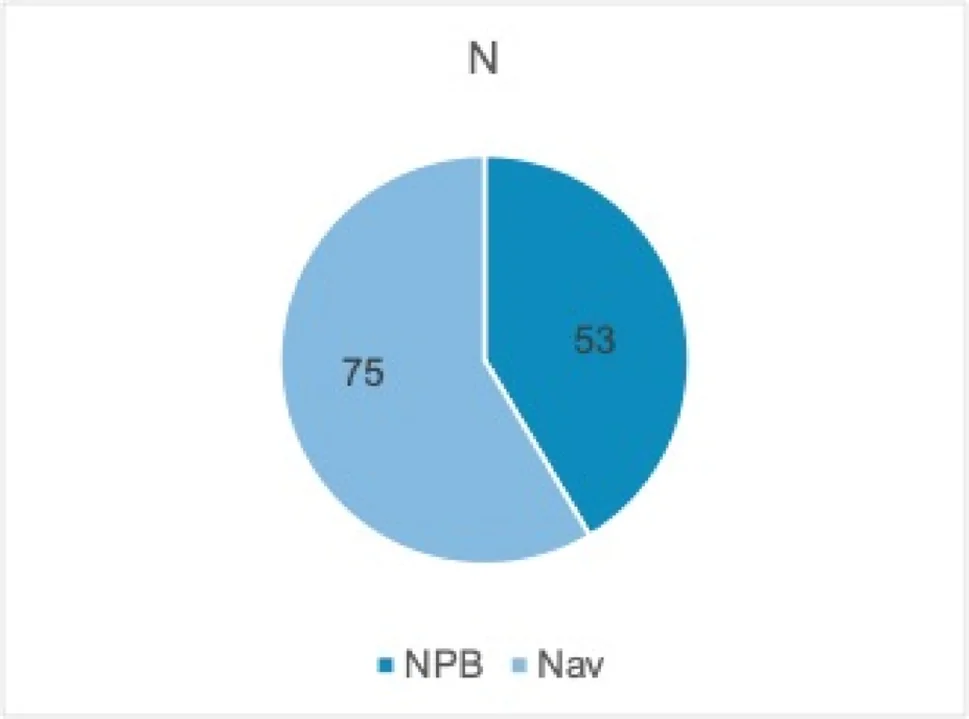
Overall Cohort

75 patients were in the Nav cohort (58.6%), and 53 in the NPB cohort (41.4%) (Figs. 1 and 2). The average age in the Nav cohort was 68.52 years and 67.19 in the NPB cohort (*P* = 0.37). The Nav cohort had 54 females (72%), and 21 males (28%), while the NPB cohort had 35 females (66%) and 18 males (34%). The ethnic distribution among each cohort was similar to the overall population, with no significant difference between the groups (*P* = 0.2007) (Table 1).

The majority of both cohorts had Stage IA lung cancer (71.9%) (Table 2). Specifically the most common subtype was stage IA2 (Table 3). Patients in the non cancer/ other category either had benign pathology or had a tumor of a non-lung primary.

**Table 2:** Clinical Stage Overall

Clinical Stage	Total	Percent
Stage 0	2	1.6%
Stage IA	92	71.9%
Stage IB	22	17.2%
Stage II	5	3.9%
Stage III-IV	2	1.6%
Non-cancer/Other	5	3.9%
Total	128	100.0%

**Table 3:** Clinical Stage by Cohort

Clinical Stage	Frozen	Nav
Stage 0	0 (0.0%)	2 (2.7%)
Stage IA1	6 (11.3%)	8 (10.7%)
Stage IA2	28 (52.8%)	25 (33.3%)
Stage IA3	11 (20.8%)	12 (16.0%)
Stage IB	1 (1.9%)	21 (28.0%)
Stage II	2 (3.8%)	3 (4.0%)
Stage III-IV	1 (1.9%)	1 (1.3%)
Non-cancer/Other	2 (3.8%)	3 (4.0%)
Total	53 (100.0%)	75 (100.0%)

**Fig. 3:**
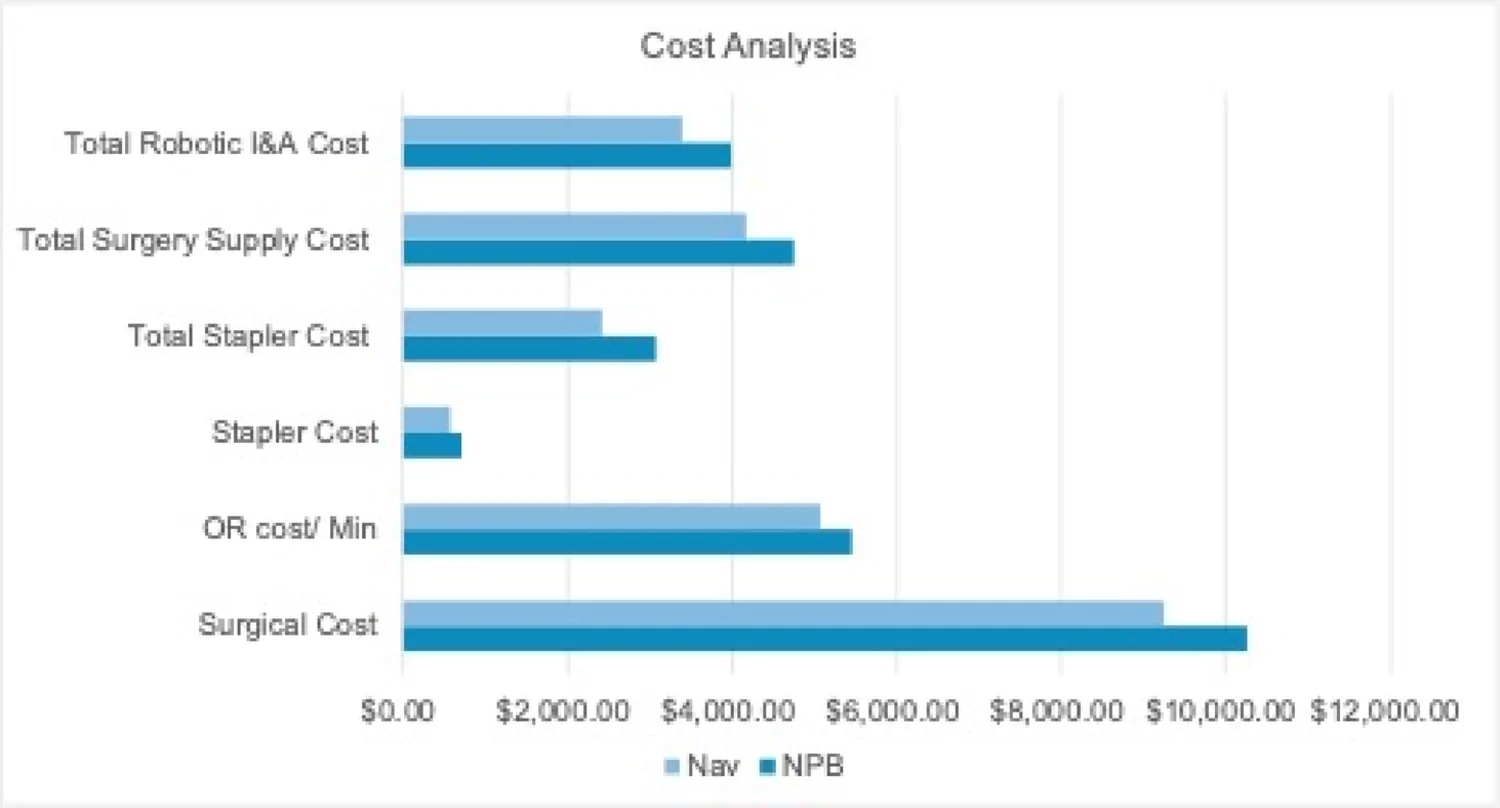
Cost Analysis

Among the NPB cohort mean surgical cost was $10,258 (1,840.42 SD). In the Nav cohort, this was $9,249.36 ($2,188.63) (*P* = 0.007) (Table 4). Operative time was 187.83 min in the NPB cohort and 174.63 in the Nav cohort (*P* = 0.161). Operative cost per minute of operative room (OR) time was higher in the NPB cohort ($5,447.08 vs. $5,063.17, *P* = 0.161). Stapler loads used were significantly higher in the NPB cohort (11.28 vs. 8.81, *P* < 0.001). Stapler cost (per # of staplers used) was also higher in the NPB cohort ($706.41) vs. Nav ($568.53) (*P* < 0.001). Factoring both previous values in, total overall stapler cost was higher in the NPB cohort compared to Nav ($3,075.85 vs. $2,422.13, *P* < 0.001). Total cost of surgical supplies was also higher in the NPB cohort ($4,749.04 vs. $4,175.39, *P* < 0.001). Overall robotic I&A cost was $3,994.30 in the NPB cohort vs. $3,395.81 in the Nav cohort (*P* < 0.001). (Table 4) In the NPB cohort, the average number of frozen specimens sent was 1.19 per case, with a mean cost of $65.35. (Fig. 3).

**Table 4:** Cost and Operative Time Analysis

Characteristic	Overall (*N* = 128)	Frozen (*N* = 53)	Nav (*N* = 75)	*p*-value
Surgery Cost	$9,667.29 ($2,104.06)	$10,258.68 ($1,840.42)	$9,249.36 ($2,188.63)	0.007
Operative time (min	180.09 (52.33)	187.83 (44.28)	174.63 (57.00)	0.161
OR Cost	$5,222.72 ($1,517.61)	$5,447.08 ($1,284.22)	$5,064.17 ($1,652.94)	0.161
Stapler Loads used	9.84 (3.20)	11.28 (3.02)	8.81 (2.94)	< 0.001
Stapler Cost ($520 per)	$625.62 ($210.03)	$706.42 ($251.76)	$568.53 ($152.29)	< 0.001
Total Stapler Cost	$2,692.81 ($837.42)	$3,075.85 ($833.92)	$2,422.13 ($731.80)	< 0.001
Total Surgery Supply Cost	$4,412.91 ($921.60)	$4,749.04 ($908.48)	$4,175.39 ($859.99)	< 0.001
Total Robotic I&A Cost	$3,643.62 ($917.02)	$3,994.30 ($922.08)	$3,395.81 ($833.92)	< 0.001

*All variables are reported as mean (SD)

## Discussion

In this retrospective study of 128 patients, we found that on average, surgical cost, operative time, robotic cost, and OR costs were higher in patients who had undergone intraoperative frozen section (NPB) compared to those with preoperative biopsy (Nav). Additionally, stapler loads, and cost were higher in this cohort. This data suggest that the use of preoperative navigational bronchoscopy may be associated with decreased intraoperative cost of lung resection.

Navigational bronchoscopy in our cohort was associated with shorter operative times, robotic, and operative costs compared to NPB patients. It is reasonable to speculate that with a clear diagnosis, surgeons can move straight to definitive lung resection without taking the time, effort and cost associated with an intraoperative wedge. The operative time was not significantly different between the cohorts however (*P* = 0.161). It is important to take this within the context of cost being determined by duration. This may not be evidence of improved efficiency. The population to which this data is applicable may be narrow as there are innumerable reasons why a patient may or may not be a candidate for either navigational bronchoscopy or diagnostic wedge resection.

Bronchoscopy also be associated with higher cost effectiveness compared to other measures. Vakil et al. found in 2021 that CT guided biopsy while least costly, was inferior to bronchoscopy when examining overall complications, time to complete procedure, rates of undetected N2-3 disease, and overall mortality [5]. This study denoted that while bronchoscopic evaluation had a higher upfront cost, less rate of complications and overall mortality may offset this when compared to CT biopsy [5]. While this did not examine Nav, these data can be compared to various others directly comparing Nav to CT guided biopsy. Such studies have found that CT guided biopsy and navigational bronchoscopy have comparable efficacy, and accuracy, with the latter demonstrating lower rates of pneumothorax [2, 6]. Taken together, these examples demonstrate that navigational bronchoscopy may also be associated with increased cost effectives when accounting for complication rates, in addition to lower operative costs found with our data. Further research is needed to compare the cost effectiveness specifically within the navigational bronchoscopy population.

Our study also found that patients in the Nav cohort had improved operative times than those in the NPB cohort (174.63 vs., 187.83, *P* = 0.161). While this measure supports the use of Nav with respect to operative efficiency, one confounding factor is the comparison of multiple vs. single anesthetic events and the cost of the navigational procedure itself. From a hospital perspective, the approach of navigational bronchoscopy then definitive resection may optimize the cost and revenue balance. Similarly, RVU credit for both procedures would be accessible due to their separation in time and would not be subject to bundling as you would see under a DRG reimbursement. For patients, however, there may be a strong incentive to only undergo one anesthetic event. However, in the current environment with the value of preoperative molecular typing and the availability of neoadjuvant strategies for even stage II disease, this immediate benefit to patients may need to be considered on balance with ideal oncologic workup.

The difference in operative times outside of cost may be valuable as prior data demonstrates increased complication rates with increasing operative times in lung resection. One cohort study in 2022 found that increased procedure duration was associated with higher risks among multiple variables [7]. They found that higher operative times were associated with higher odds of intraoperative red blood cell transfusion (*P*<0.001) and increased length of stay (*P* < 0.001) in both thoracotomies and minimally invasive lobectomies [7]. A study in 2022 demonstrated similar findings when only examining thoracoscopic lobectomy [8]. It is evident based on our data and these listed studies that increased operative time is not only associated with higher complication rates, but also higher cost. Based on our institution, an operative minute costs $29, and as such the NPB cohort had an average cost of $5,447.08 compared to $5,064.17 in the Nav cohort (*P* = 0.161). Overall, our data and the highlighted research underscore the importance of decreased operative times with respect to patient complications and cost.

While our data demonstrate an improved operative and robotic utilization cost in patients who had a navigational bronchoscopy, this method demonstrates other benefits with respect to clinical yield. Namely, navigational bronchoscopy may be able to access lesions previously deemed too small or peripheral for a traditional approach. Electromagnetic navigational bronchoscopy has been found to have good sensitivity (pooled 77%) and an acceptable safety profile for the diagnosis of peripheral pulmonary lesions [10, 11]. This is contrast to previously cited yield of traditional bronchoscopy in such lesions, which is 34% of lesions < 2 cm[12]. In our study, we do recognize that intraoperative wedge resection may have been selected in certain patients due to nodule size and location. This difference may become less significant in the future with increasing complexity of the navigational platform.

There are several limitations to this study. As this is a single institution retrospective study, the results are difficult to generalize. There may be inherent selection bias as navigational bronchoscopy has increased utilization in more recent years. Additionally, when comparing the cohorts we may be comparing nodules which are able to be accessed by navigational bronchoscopy vs. those that are not. In the latter group, there may be smaller and more peripheral nodules which are more amenable to wedge resection. Operative time also does not take into account whether further dissection or vascular isolation was performed while awaiting frozen section data, which can contribute to an overall decreased operative time as well. We are also comparing a single anesthetic event to multiple, which ignores the other cost events in each patient’s episode of care, including the cost of the navigational bronchoscopy. We are additionally unable to determine hospital reimbursement costs for these procedures, which may also favor the usage of one method. Additionally, we did not factor in the overhead cost of the navigational bronchoscopy system, and the cost of that individual procedure for each patient. This will affect the cost effectiveness comparison. We also did not examine patient reported or long term outcomes. When examining similar data, Wei et al. noted that robotic assisted surgery compared to VATS had reduced symptoms and better clinical outcomes at 1 year, but at higher costs with utilization of the robotic platform [9]. It is critical to examine this data in this context.

In our study, we found that patients undergoing preoperative navigational bronchoscopy had reduced operative costs and time of their lung resection when compared with those undergoing intraoperative biopsy. This data suggests that the use of preoperative navigational bronchoscopy is associated with decreased cost of robotic lung resection. We found statistically significant differences with respect to stapler loads used, stapler individual and total cost, surgical supply coat, and robotic I&A cost. This encourages the use of this platform with respect to reducing intraoperative costs and operative duration in lung resection. Further research and data collection is needed to understand the implications this has on clinical practice.

## Data Availability

No datasets were generated or analysed during the current study.

## Abbreviations

Nav: Navigational bronchoscopy
NPB: No preoperative biopsy
I&A: Instruments and accessories

## References

[1] Marino KA, Sullivan JL, Weksler B (2016) Electromagnetic Navigation Bronchoscopy for Identifying Lung Nodules for Thoracoscopic Resection. Ann Thorac Surg 102(2):454–457. 10.1016/j.athoracsur.2016.03.010 27173068

[2] Tan Y, Shen S, Wang C, Zhou Q, Jing Q (2024) Comparison of electromagnetic navigation bronchoscopy localization and CT-guided percutaneous localization in resection of lung nodules: A protocol for systematic review and meta-analysis. Medicine 103(38):e39760. 10.1097/MD.0000000000039760 PMC11419552 39312306

[3] Marchevsky AM, Changsri C, Gupta I, Fuller C, Houck W, McKenna RJ (2004) Frozen Section Diagnoses of Small Pulmonary Nodules: Accuracy and Clinical Implications. Ann Thorac Surg 78(5):1755–1759. 10.1016/j.athoracsur.2004.05.003 15511468

[4] Liu S, Wang R, Zhang Y et al (2016) Precise Diagnosis of Intraoperative Frozen Section Is an Effective Method to Guide Resection Strategy for Peripheral Small-Sized Lung Adenocarcinoma. JCO 34(4):307–313. 10.1200/JCO.2015.63.4907 26598742

[5] Vakil E, Jackson N, Sainz-Zuñega PV et al (2021) Optimizing Diagnostic and Staging Pathways for Suspected Lung Cancer. Chest 160(6):2304–2323. 10.1016/j.chest.2021.06.065 34256049

[6] Kuo SW, Tseng YF, Dai KY, Chang YC, Chen KC, Lee JM (2019) Electromagnetic Navigation Bronchoscopy Localization versus Percutaneous CT-Guided Localization for Lung Resection via Video-Assisted Thoracoscopic Surgery: A Propensity-Matched Study. JCM 8(3):379. 10.3390/jcm8030379 PMC6463081 30889927

[7] Dexter E, Attwood K, Demmy T, Yendamuri S (2022) Does Operative Duration of Lobectomy for Early Lung Cancer Increase Perioperative Morbidity? Ann Thorac Surg 114(3):941–947. 10.1016/j.athoracsur.2022.01.040 PMC9381650 35183506

[8] Gómez-Hernández MT, Forcada C, Varela G et al (2022) Operating time: an independent and modifiable risk factor for short-term complications after video-thoracoscopic pulmonary lobectomy. Eur J Cardiothorac Surg 62(6):ezac503. 10.1093/ejcts/ezac503 36264130

[9] Wei X, Yu H, Dai W et al (2026) Long-Term Patient-Reported Outcomes after Multiport Robot-Assisted Surgery versus Video-Assisted Surgery for Lung Cancer: An Observational Cohort Study. Ann Surg Oncol 33(5):4288–4300. 10.1245/s10434-025-18834-0 41665783

[10] Folch EE, Labarca G, Ospina-Delgado D et al (2020) Sensitivity and Safety of Electromagnetic Navigation Bronchoscopy for Lung Cancer Diagnosis. Chest 158(4):1753–1769. 10.1016/j.chest.2020.05.534 32450240

[11] Lentz RJ, Frederick-Dyer K, Planz VB et al (2025) Navigational Bronchoscopy or Transthoracic Needle Biopsy for Lung Nodules. N Engl J Med 392(21):2100–2112. 10.1056/NEJMoa2414059 PMC12640718 40387025

[12] Bai C, Choi CM, Chu CM et al (2016) Evaluation of Pulmonary Nodules. Chest 150(4):877–893. 10.1016/j.chest.2016.02.650 26923625

